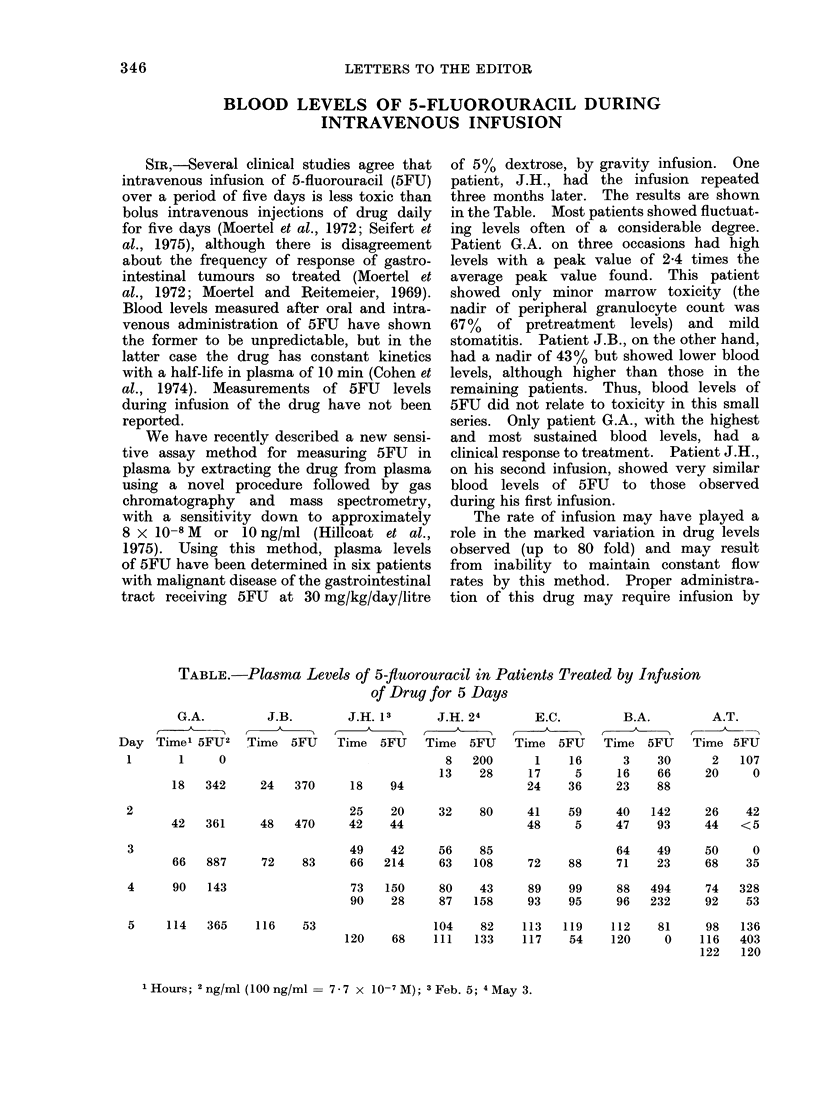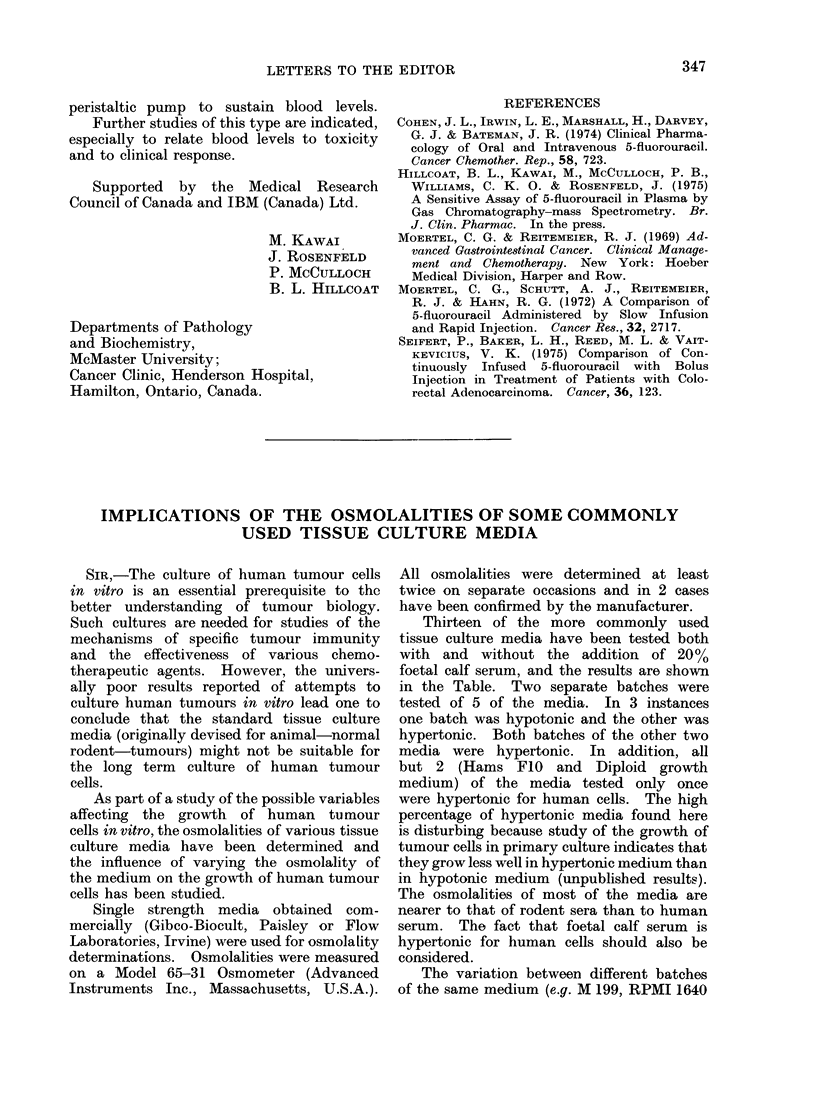# Letter: Blood levels of 5-fluorouracil during intravenous infusion.

**DOI:** 10.1038/bjc.1976.51

**Published:** 1976-03

**Authors:** M. Kawai, J. Rosenfeld, P. McCulloch, B. L. Hillcoat


### BLOOD LEVELS OF 5-FLUOROURACIL DURING INTRAVENOUS INFUSION

SIR,—Several clinical studies agree that intravenous infusion of 5-fluorouracil (5FU) over a period of five days is less toxic than bolus intravenous injections of drug daily for five days (Moertel *et al.*, 1972; Seifert *et al.*, 1975), although there is disagreement about the frequency of response of gastro-intestinal tumours so treated (Moertel *et al.*, 1972; Moertel and Reitemeier, 1969). Blood levels measured after oral and intravenous administration of 5FU have shown the former to be unpredictable, but in the latter case the drug has constant kinetics with a half-life in plasma of 10 min (Cohen *et al.*, 1974). Measurements of 5FU levels during infusion of the drug have not been reported.

We have recently described a new sensitive assay method for measuring 5FU in plasma by extracting the drug from plasma using a novel procedure followed by gas chromatography and mass spectrometry, with a sensitivity down to approximately $8 \times 10^{-8}$ M or 10 ng/ml (Hillcoat *et al.*, 1975). Using this method, plasma levels of 5FU have been determined in six patients with malignant disease of the gastrointestinal tract receiving 5FU at 30 mg/kg/day/litre of 5% dextrose, by gravity infusion. One patient, J.H., had the infusion repeated three months later. The results are shown in the Table. Most patients showed fluctuating levels often of a considerable degree. Patient G.A. on three occasions had high levels with a peak value of 2·4 times the average peak value found. This patient showed only minor marrow toxicity (the nadir of peripheral granulocyte count was 67% of pretreatment levels) and mild stomatitis. Patient J.B., on the other hand, had a nadir of 43% but showed lower blood levels, although higher than those in the remaining patients. Thus, blood levels of 5FU did not relate to toxicity in this small series. Only patient G.A., with the highest and most sustained blood levels, had a clinical response to treatment. Patient J.H., on his second infusion, showed very similar blood levels of 5FU to those observed during his first infusion.

The rate of infusion may have played a role in the marked variation in drug levels observed (up to 80 fold) and may result from inability to maintain constant flow rates by this method. Proper administration of this drug may require infusion by

TABLE.—*Plasma Levels of 5-fluorouracil in Patients Treated by Infusion of Drug for 5 Days*

| | G.A. | | J.B. | | J.H. 1[3] | | J.H. 2[4] | | E.C. | | B.A. | | A.T. | |
|---|---|---|---|---|---|---|---|---|---|---|---|---|---|---|
| Day | Time[1] | 5FU[2] | Time | 5FU | Time | 5FU | Time | 5FU | Time | 5FU | Time | 5FU | Time | 5FU |
| 1 | 1 | 0 | | | | | 8 | 200 | 1 | 16 | 3 | 30 | 2 | 107 |
| | | | | | | | 13 | 28 | 17 | 5 | 16 | 66 | 20 | 0 |
| | 18 | 342 | 24 | 370 | 18 | 94 | | | 24 | 36 | 23 | 88 | | |
| 2 | | | | | 25 | 20 | 32 | 80 | 41 | 59 | 40 | 142 | 26 | 42 |
| | 42 | 361 | 48 | 470 | 42 | 44 | | | 48 | 5 | 47 | 93 | 44 | <5 |
| 3 | | | | | 49 | 42 | 56 | 85 | | | 64 | 49 | 50 | 0 |
| | 66 | 887 | 72 | 83 | 66 | 214 | 63 | 108 | 72 | 88 | 71 | 23 | 68 | 35 |
| 4 | 90 | 143 | | | 73 | 150 | 80 | 43 | 89 | 99 | 88 | 494 | 74 | 328 |
| | | | | | 90 | 28 | 87 | 158 | 93 | 95 | 96 | 232 | 92 | 53 |
| 5 | 114 | 365 | 116 | 53 | | | 104 | 82 | 113 | 119 | 112 | 81 | 98 | 136 |
| | | | | | 120 | 68 | 111 | 133 | 117 | 54 | 120 | 0 | 116 | 403 |
| | | | | | | | | | | | | | 122 | 120 |

[1] Hours; [2] ng/ml (100 ng/ml = $7 \cdot 7 \times 10^{-7}$ M); [3] Feb. 5; [4] May 3.

peristaltic pump to sustain blood levels.

Further studies of this type are indicated, especially to relate blood levels to toxicity and to clinical response.

Supported by the Medical Research Council of Canada and IBM (Canada) Ltd.

M. KAWAI
J. ROSENFELD
P. MCCULLOCH
B. L. HILLCOAT

Departments of Pathology
and Biochemistry,
McMaster University;
Cancer Clinic, Henderson Hospital,
Hamilton, Ontario, Canada.

REFERENCES

COHEN, J. L., IRWIN, L. E., MARSHALL, H., DARVEY, G. J. & BATEMAN, J. R. (1974) Clinical Pharmacology of Oral and Intravenous 5-fluorouracil. *Cancer Chemother. Rep.*, **58**, 723.

HILLCOAT, B. L., KAWAI, M., MCCULLOCH, P. B., WILLIAMS, C. K. O. & ROSENFELD, J. (1975) A Sensitive Assay of 5-fluorouracil in Plasma by Gas Chromatography–mass Spectrometry. *Br. J. Clin. Pharmac.* In the press.

MOERTEL, C. G. & REITEMEIER, R. J. (1969) *Advanced Gastrointestinal Cancer. Clinical Management and Chemotherapy.* New York: Hoeber Medical Division, Harper and Row.

MOERTEL, C. G., SCHUTT, A. J., REITEMEIER, R. J. & HAHN, R. G. (1972) A Comparison of 5-fluorouracil Administered by Slow Infusion and Rapid Injection. *Cancer Res.*, **32**, 2717.

SEIFERT, P., BAKER, L. H., REED, M. L. & VAITKEVICIUS, V. K. (1975) Comparison of Continuously Infused 5-fluorouracil with Bolus Injection in Treatment of Patients with Colorectal Adenocarcinoma. *Cancer*, **36**, 123.